# Outcomes of Multimodal Treatment in Elderly Patients with Localized Non-Small Lung Cancer from a Radiation Oncology Point of View: Special Focus on Low-Dose Cisplatin

**DOI:** 10.3390/cancers16020327

**Published:** 2024-01-11

**Authors:** Niklas Josua Alt, Julian Muster, David Alexander Ziegler, Stephanie Bendrich, Sandra Donath, Andrea Hille, Mahalia Zoe Anczykowski, Carla Marie Zwerenz, Friederike Braulke, Alexander von Hammerstein-Equord, Tobias Raphael Overbeck, Hannes Treiber, Manuel Guhlich, Rami El Shafie, Stefan Rieken, Martin Leu, Leif Hendrik Dröge

**Affiliations:** 1 Department of Radiotherapy and Radiation Oncology, University Medical Center Göttingen, Robert-Koch-Str. 40, 37075 Göttingen, Germany; niklasjosua.alt@stud.uni-goettingen.de (N.J.A.); alexander.ziegler@med.uni-goettingen.de (D.A.Z.); stephanie.bendrich@med.uni-goettingen.de (S.B.); sandra.donath@med.uni-goettingen.de (S.D.); ahille@med.uni-goettingen.de (A.H.); mahalia-zoe.anczykowski@med.uni-goettingen.de (M.Z.A.); carlamarie.zwerenz@med.uni-goettingen.de (C.M.Z.); manuel.guhlich@med.uni-goettingen.de (M.G.); rami.elshafie@med.uni-goettingen.de (R.E.S.); stefan.rieken@med.uni-goettingen.de (S.R.); martin.leu@med.uni-goettingen.de (M.L.); 2 Göttingen Comprehensive Cancer Center (G-CCC), University Medical Center Göttingen, Von-Bar-Str. 2/4, 37075 Göttingen, Germany; friederike.braulke@med.uni-goettingen.de (F.B.); alexander.hammerstein@med.uni-goettingen.de (A.v.H.-E.); tobias.overbeck@med.uni-goettingen.de (T.R.O.); hannes.treiber@med.uni-goettingen.de (H.T.); 3 Department of Cardio-Thoracic and Vascular Surgery, University Medical Center Göttingen, Robert-Koch-Str. 40, 37075 Göttingen, Germany; 4 Department of Hematology and Medical Oncology, University Medical Center Göttingen, Robert-Koch-Str. 40, 37075 Göttingen, Germany

**Keywords:** non-small cell lung cancer, radiotherapy, radiochemotherapy, multimodal therapy, elderly, low-dose cisplatin, cisplatin/vinorelbine, toxicity, outcomes, clinical characteristics

## Abstract

**Simple Summary:**

Identification of the optimal treatment strategy is challenging in elderly with non-small cell lung cancer (NSCLC). Outcomes (1) in elderly vs. younger patients and (2) with low-dose cisplatin vs. cisplatin/vinorelbine were studied. Elderly included more males, had a lower Karnofsky index, more comorbidities, and lower stages. Low-dose cisplatin patients had higher age, more comorbidities, and lower stages. We observed reduced dermatitis and dysphagia and increased anemia and thrombocytopenia in elderly, without increased ≥grade 3 toxicities. Low-dose cisplatin was less toxic than cisplatin/vinorelbine. Survival was lower in elderly vs. younger and comparable between both chemotherapy protocols. In elderly, gender, Karnofsky index, stage, and multimodal treatment (including additional surgery/systemic therapy) were prognostic factors. In elderly, we found acceptable toxicities with radiotherapy but the need for the improvement of outcomes. Multimodal strategies showed a favorable prognosis and can reasonably be considered. Low-dose cisplatin should be discussed on an individual basis due to favorable toxicity and outcomes.

**Abstract:**

Identification of the optimal treatment strategy is challenging in elderly with localized non-small cell lung cancer (NSCLC). Concurrent chemotherapy with low-dose cisplatin represents an option for elderly. Outcomes (1) in elderly (≥70 years, *n* = 158) vs. younger patients (*n* = 188) and (2), independently of age, in definitive radiochemotherapy, with low-dose cisplatin (*n* = 125) vs. cisplatin/vinorelbine (*n* = 76) were studied. Elderly included more males, had a lower Karnofsky index, more comorbidities, and lower stages. Low-dose cisplatin patients (vs. cisplatin/vinorelbine) had higher age, more comorbidities, and lower stages. We observed reduced dermatitis and dysphagia and increased anemia and thrombocytopenia in elderly vs. younger patients, without increased ≥grade 3 toxicities. Low-dose cisplatin was less toxic than cisplatin/vinorelbine. Survival outcomes were lower in elderly vs. younger and comparable between low-dose cisplatin and cisplatin/vinorelbine. In elderly, gender, Karnofsky index, stage, and multimodal treatment (including additional surgery/systemic therapy) were identified as prognostic factors. In conclusion, we found evidence for an acceptable toxicity profile and the need for improvement of outcomes in elderly with localized NSCLC. Multimodal strategies (including additional surgery/systemic treatment) showed favorable outcomes and should be reasonably considered in elderly who are deemed fit enough. Low-dose cisplatin should be discussed on an individual basis due to favorable toxicity and outcomes.

## 1. Introduction

In the European Union, in 2018, the observed lung cancer-related numbers of death were approximately 76,000 in women and 161,000 in men [1]. The predicted numbers of death for 2023 are approximately 84,000 in women and 159,000 in men [1]. In Germany, the median age at lung cancer diagnosis is 69 years in women and 70 years in men [2]. Generally, there is a change in population towards older ages [3]. Higher age is associated with an increased risk of lung cancer [3]. In a patterns of care study on lung cancer across Europe by Sant et al., 46.9% of the patients were ≥70 years old [4].

At the same time, when considering anti-cancer treatment, there are relevant differences between elderly and younger patients [3]. These differences include physiologic changes in the respiratory system (e.g., reduction in chest wall compliance, osteoporosis, vertebral stiffness, and rib calcification) or further organ systems (e.g., reduced renal function, aging of the immune system) [3,5,6]. Additionally, elderly present with decreased pulmonary function and increased risk for pulmonary infections [3,7]. There is evidence that, in elderly patients, treatment-related toxicities (e.g., after application of cisplatin) can be increased [8].

Remarkably, in spite of the high percentage of elderly patients with lung cancer and, at the same time, age-specific characteristics, these patients are clearly under-represented in clinical trials [9]. Tang et al. studied the putative eligibility for clinical trials in patients with lung cancer at the age of ≥65 years. They found that >50% of these patients were not suitable [9]. In developed countries, the usual cut-off to consider patients to be ‘elderly’ is 70 years [3,10,11]. Bonanno et al. emphasize that, especially for non-small cell lung cancer (NSCLC), the increasing focus on multimodal strategies requires personalized treatment and the consideration of toxicity risks in elderly patients [12].

Additionally, definitive treatment with concurrent radiochemotherapy (RCT) is associated with relevant risks of toxicities [13]. During the last decades, different chemotherapy protocols were introduced [14]. According to the German S3 guidelines for lung cancer, in NSCLC, most studies applied cisplatin-containing combination protocols (e.g., cisplatin and vinorelbine [15]) [16] (p. 192). The guidelines emphasize that general condition and comorbidities have to be considered for treatment selection [16] (p. 192). Low-dose cisplatin (as described by Schaake-Koning et al. before [17]) was discussed as an alternative option with potentially less toxicity risks in elderly or frail patients [18].

Here, we present a retrospective study on patients with localized NSCLC from a radiation oncology point of view. Patients were included when curative radiotherapy (RT) or RCT (neoadjuvant RT/RCT before surgery, adjuvant RT/RCT after surgery, or definitive RT/RCT) were applied at our University Medical Center, where the certified lung cancer center was initiated in 2009. We compared clinical characteristics, treatment characteristics, toxicities, and outcomes in younger (<70 years) vs. elderly (≥70 years) patients. Additionally, we studied prognostic factors in elderly. Furthermore, we compared clinical characteristics, treatment characteristics, toxicities, and outcomes in definitive RCT (independently from age) with cisplatin/vinorelbine vs. low-dose cisplatin.

## 2. Patients and Methods

### 2.1. Study Design

Patient records were screened for NSCLC and RT. The treatment period from 01/2008 to 12/2019 was considered. The general inclusion criteria for this project were as follows: RT in curative intent (neoadjuvant RT/RCT before surgery, adjuvant RT/RCT after surgery, or definitive RT/RCT) and conventional dose fractionation (1.8–2.0 Gy). The exclusion criteria were as follows: UICC stage IV, planned total RT dose of <50 Gy, and Durvalumab consolidation based on the PACIFIC trial [19]. The study was approved by the local ethics committee (University Medical Center Göttingen, no. 2/8/20). In total, 749 patients were identified in medical records. In accordance with the mentioned criteria, 346 patients were suitable for further analyses.

**Figure 1:**
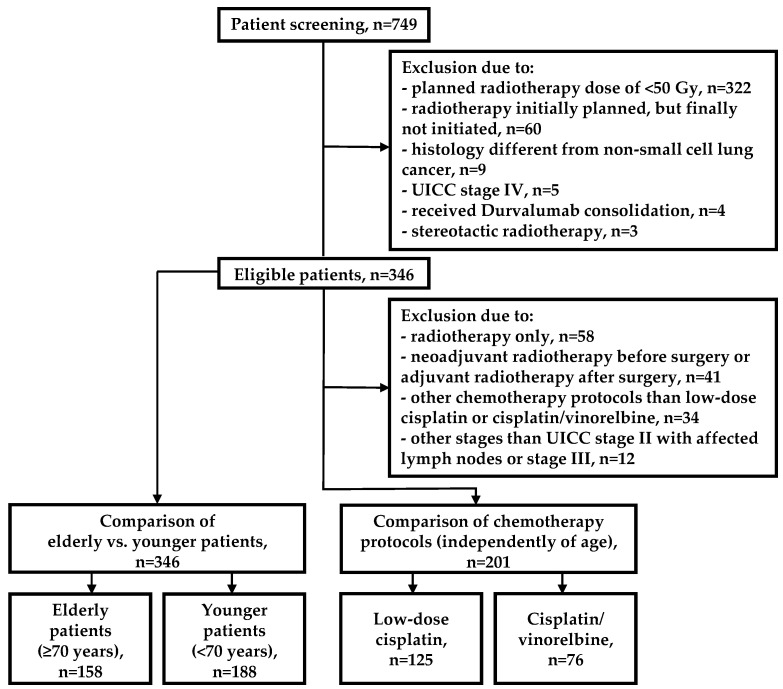
Flow chart with information on patient inclusion.

In the first part of the study (Figure 1, left side), for the comparison of elderly vs. younger patients, no further criteria were defined. Here, we considered all the 346 patients (≥70 years, *n* = 158, <70 years, *n* = 188).

In the second part of the study (Figure 1, right side), we present a comparison of the chemotherapy protocols (independently of age). Here, further inclusion criterion was the following: UICC stage II with affected lymph nodes or stage III (indications for concurrent chemotherapy according to NCCN guidelines [20]). Further exclusion criteria were as follows: RT only, neoadjuvant RT before surgery or adjuvant RT after surgery, and application of other chemotherapy protocols than low-dose cisplatin or cisplatin/vinorelbine.

### 2.2. Radiochemotherapy

Patients received radiotherapy at the University Medical Center. The lung cancer center was initiated in 2009 and is certified since 2014 by the German Cancer Society (Deutsche Krebsgesellschaft, DKG, Berlin, Germany). Patients were evaluated in the multidisciplinary tumor board. Diagnostic and treatment procedures were initiated in accordance with the national and international guidelines [16,20]. In RT planning, the system Eclipse (Varian Medical Systems, Palo Alto, California, USA) was used. RT target volumes were defined in accordance with the respective guidelines [21]. RT was applied with Varian linear accelerators and photon energies of 6 MeV or 20 MeV. The image guidance included electronic portal imaging device (EPID), on-board-kV-imaging (OBI), and cone-beam-CT (CBCT). In definitive treatment, CT scans were standardly carried out after application of 20 Gy and 40 Gy for treatment monitoring. Systemic treatment was regularly applied on an inpatient basis, either by the radiotherapy department or by cooperation partners within the lung cancer center. In definitive RCT, standard concurrent chemotherapy protocols consisted of cisplatin/vinorelbine (2 cycles of cisplatin (20 mg/m^2^ of body surface area/d, d 1–4, q4 w) and vinorelbine (orally, 50 mg/m^2^, or intravenously, 20 mg/m^2^ of body surface area/d, days 1, 8, 15)) [15] and low-dose ciplatin (as described by Schaake-Koning et al., application of 6 mg/m^2^ of body surface area on each day with radiotherapy application [17]). The treatment decision was made individually, based on performance status and comorbidities. Due to the possibly favorable toxicity profile [18], low-dose cisplatin was considered as an alternative option especially in elderly or frail patients. During RT or RCT, patients were monitored at least weekly for toxicity monitoring (clinical evaluation, blood samples). Patients received a CT scan at 6 weeks after the end of treatment for response evaluation and evaluation of side effects (i.e., presence of pneumonitis). In the radiotherapy department, patients were planned for follow-up at least annually. Additionally, follow-up examinations were performed by the cooperation partners (i.e., internists, pulmonologists, oncologists).

### 2.3. Endpoints and Statistical Analysis

Toxicities were scored in accordance with the Common Terminology Criteria for Adverse Events during the whole follow-up period [22]. For analysis, the tumor stages were documented based on the information in the medical records and, thus, reflect each current classification of the study period (2008–2019, 6th, 7th, and 8th edition of TNM/UICC/AJCC staging systems). If additional classification was necessary after extraction of the information from the medical records, the current 8th edition of the staging system was used [23,24,25,26]. The survival endpoints were overall survival (OS, patient death), progression-free survival (PFS, patient death or locoregional/distant recurrence), locoregional progression-free survival (LPFS, patient death or locoregional recurrence), and distant progression-free survival (DPFS, patient death or distant recurrence). Survival times were calculated from the date of diagnosis. For data administration and statistical analysis, the programs Statistica (v13.3, TIBCO Software Inc., Palo Alto, California, USA), Microsoft Excel (v2016, Microsoft Corporation, Redmond, Washington, DC, USA), and SPSS (v27, IBM Corp., Armonk, NY, USA) were used. For illustration of survival curves, the Software R (v4.1.0) with the plugin KMWin (v1.53) was used [27]. In statistical analysis, a *p*-value of <0.05 was considered statistically significant.

## 3. Results

### 3.1. Clinical and Treatment Characteristics

#### 3.1.1. Elderly vs. Younger Patients

The median ages of the elderly and younger cohort were 75.8 and 61.2 years (Table 1). Elderly patients presented with more males, shorter-follow-up, lower Karnofsky index, higher Charlson comorbidity index, and lower UICC stages (comparison of stage IIA-IIIA vs. IIIB-IIIC). Radiotherapy was completed as planned in 288/346 patients (83.2%). Elderly patients were treated with RT only more often, whereas bimodality and trimodality therapies were carried out less frequently. In elderly patients with definitive treatment, RCT (vs. RT only) was less often carried out. In case of concurrent RCT, as clinically indicated, elderly patients received more often low-dose cisplatin, whereas younger patients were more likely to be treated with cisplatin/vinorelbine. In total, only 20 elderly patients received cisplatin/vinorelbine. Please see Table 1 for further details.

#### 3.1.2. Cisplatin/Vinorelbine vs. Low-Dose Cisplatin in Definitive Radiochemotherapy (Independently of Age)

Results of definitive RCT with low-dose cisplatin vs. cisplatin/vinorelbine were investigated without taking patients’ age into account. The results reflect strategies in multidisciplinary tumor board and in clinical routine. Patients treated with low-dose cisplatin (vs. cisplatin/vinorelbine) had higher age (median 68.5 vs. 62.6 years) and more comorbidities (median Charlson comorbidity index 6 vs. 5). Additionally, patients with low-dose cisplatin presented with lower nodal stages (N0–1 vs. N2–3). Concomitant chemotherapy was completed more frequently in patients with low-dose cisplatin (low-dose cisplatin, 78.4% vs. cisplatin/vinorelbine, 64.5%, *p* = 0.03). Please see Table 2 for further details. When comparing RCT with cisplatin/vinorelbine vs. low-dose cisplatin, the determination of specific chemotherapy-related toxicities is of valid interest (see Section 3.2.2). Therefore, we compared patients who received definitive RT only (*n* = 58, exclusion for the actual goals of the presented study, please see flow chart in Figure 1) vs. patients who received RCT with cisplatin/vinorelbine or with low-dose cisplatin (Supplementary Table S1). Again, the results presented here reflect strategies recommended in multidisciplinary tumor boards and in clinical routine. Main reasons for omission of chemotherapy included patient age, reduced general condition (except reduced performance status because of high tumor burden), or relevant comorbidities. Thus, among other differences, patients who received RT only presented with higher age, lower Karnofsky index, and higher Charlson comorbidity index (Supplementary Table S1).

### 3.2. Toxicities

#### 3.2.1. Elderly vs. Younger Patients

In elderly patients, when compared with younger patients, dermatitis and dysphagia (≥grade 1) occurred less frequently. Furthermore, in elderly patients, anemia (≥grade 1) and thrombocytopenia (≥grade 1) occurred significantly more often. The grade 4 toxicities included leukopenia (*n* = 20), dyspnea (*n* = 9), thrombocytopenia (*n* = 3), anemia (*n* = 1), and pneumonitis (*n* = 1). In 2 patients, grade 5 was documented for dyspnea. Please see Table 3 for further details.

#### 3.2.2. Cisplatin/Vinorelbine vs. Low-Dose Cisplatin in Definitive Radiochemotherapy (Independently of Age)

In patients treated with low-dose cisplatin, when compared to cisplatin/vinorelbine, dysphagia (≥grades 2 and 3) occurred less frequently. Additionally, with low-dose cisplatin, the rates of nausea (≥grades 1 and 2) were lower. Patients with low-dose cisplatin were less frequently affected by leukopenia (≥grades 1, 2, and 3). Please see Table 4 for further details. The grade 4 toxicities included pneumonitis (*n* = 1), dyspnea (*n* = 5), leukopenia (*n* = 15), and thrombocytopenia (*n* = 2). In 1 patient, grade 5 dyspnea was documented.

We found significant differences between low-dose cisplatin and cisplatin/vinorelbine for dysphagia, nausea, and leukopenia. It is of valid interest whether these toxicities can be determined as specific toxicities of chemotherapy in the presented study. Therefore, we compared patients who received definitive RT only (*n* = 58, exclusion for the actual goals of the presented study, please see flow chart in Figure 1) vs. patients who received RCT with cisplatin/vinorelbine or with low-dose cisplatin. Among other differences, dysphagia (RT only vs. cisplatin/vinorelbine), nausea (RT only vs. cisplatin/vinorelbine), and leukopenia (RT only vs. low-dose cisplatin and RT only vs. cisplatin/vinorelbine) occurred more frequently with chemotherapy (Supplementary Table S2). Thus, for these toxicity parameters, there is evidence that differences between patients who received cisplatin/vinorelbine vs. low-dose cisplatin are specifically related to applied chemotherapy protocols.

### 3.3. Outcomes

#### 3.3.1. Elderly Patients

##### Elderly vs. Younger Patients

**Figure 2:**
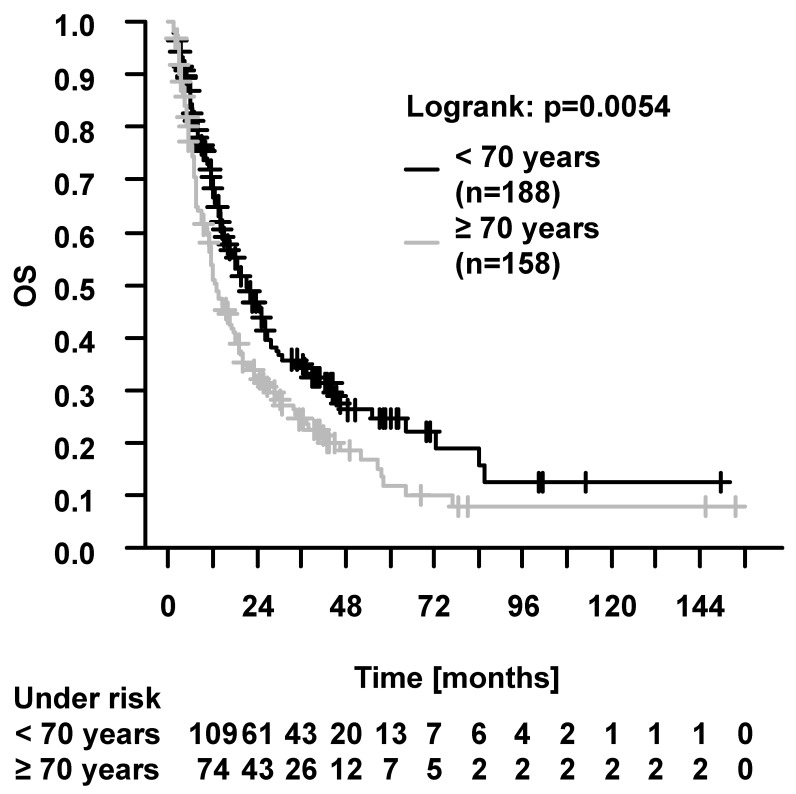
Overall survival (OS) in elderly (≥70 years) vs. younger (<70 years) patients. OS was significantly better in younger patients (*p* = 0.0054, log-rank test). The 2-year and 5-year OS were 33.0% vs. 45.8% and 11.8% vs. 24.8%, respectively.

In the whole cohort (elderly and younger patients), the 2-year and 5-year OS were 39.9% and 18.6%, respectively. The 2-year and 5-year PFS were 25.6% and 10.5%, respectively. When comparing elderly vs. younger patients (log-rank test), there was a significant difference in OS (Figure 2), but not in PFS (5 years, elderly, 7.9%vs. younger, 12.6%, *p* = 0.48), LPFS (5 years, elderly, 8.0% vs. younger, 14.5%, *p* = 0.13), and DPFS (5 years, elderly, 9.8% vs. younger, 16.6%, *p* = 0.2). We performed univariable Cox regression analysis including additional parameters with possible influence on survival. Multivariable analysis was performed using parameters with influence on survival in univariable analysis (*p* < 0.05) and representation of all patients. In multivariable analysis, patient age (elderly vs. younger patients) was identified as a prognostic factor only for OS, but not for PFS, LPFS, and DPFS (please see Supplementary Tables S3 and S4 for analysis including further parameters). Events for PFS (i.e., patient death or locoregional/distant recurrence) occurred in 133/158 (84.2%) elderly patients and in 154/188 (81.9%) younger patients. Tumor progression was documented in 40/158 (25.3%) elderly patients and in 92/188 (48.9%) younger patients. Locoregional recurrences occurred in 31/158 (19.6%) elderly patients and in 65/188 (34.6%) younger patients. Distant recurrences were documented in 22/158 (13.9%) elderly patients and in 60/188 (31.9%) younger patients.

##### Prognostic Factors in Elderly Patients

**Figure 3:**
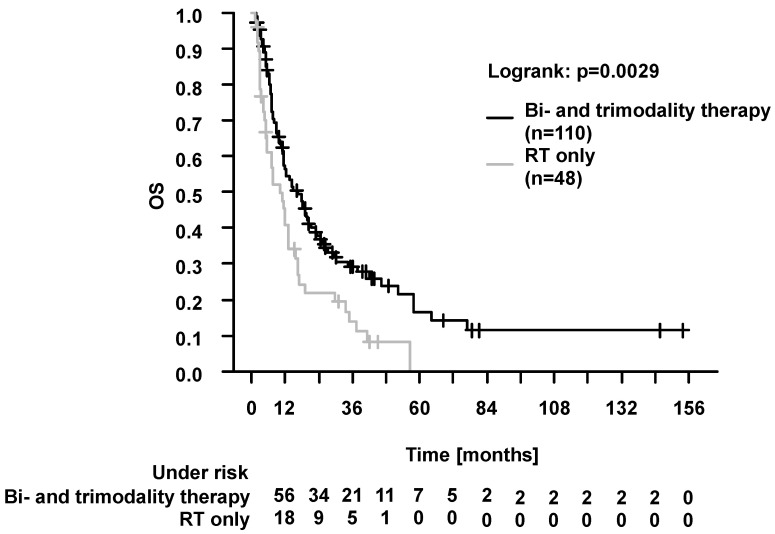
Overall survival (OS) in elderly (≥70 years) patients, radiotherapy (RT) only vs. bimodality and trimodality therapy. OS was significantly better in patients with bi-and trimodality therapy (*p* = 0.0029, log-rank test). The 2-year and 5-year OS were 21.9% vs. 37.8% and 0.0% vs. 16.6%, respectively.

For analysis of prognostic factors in elderly patients (*n* = 158), first, univariable Cox regression analysis was carried out. Gender, T stage, histology (adenocarcinoma vs. other histology), and treatment concept (RT only vs. bi- and trimodality therapy) were identified as prognostic factors for OS, PFS, LPFS, and DPFS (each, *p* < 0.05; Supplementary Table S5; Figure 3). Multivariable analysis was performed using parameters with influence on survival in univariable analysis (*p* < 0.05) and representation of all patients ≥70 years. Here, gender, T stage, treatment concept, and Karnofsky index had a significant influence on survival (*p* < 0.05 for OS, PFS, LPFS and DPFS; exception: *p* = 0.07 for T stage and LPFS). For histology, the significance was lost in multivariable analysis. Please see Table 5 for details.

#### 3.3.2. Cisplatin/Vinorelbine vs. Low-Dose Cisplatin in Definitive Radiochemotherapy (Indepently of Age)

**Figure 4:**
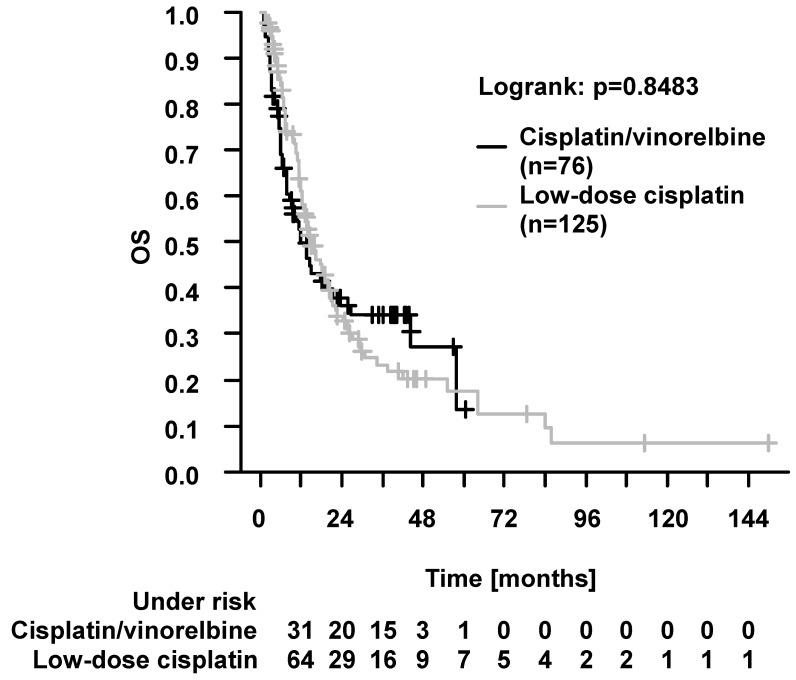
Overall survival (OS) in patients who received definitive radiochemotherapy with cisplatin/vinorelbine vs. low-dose cisplatin (independently of age). There were no differences in OS (*p* = 0.8483, log-rank test). The 2-year and 5-year OS were 37.9% vs. 33.9% and 13.6% vs. 17.6%, respectively.

There were no differences in OS (Figure 4, 2-year and 5-year, 37.9% vs. 33.9% and 13.6% vs. 17.6%, *p* = 0.8483), PFS (2-year and 5-year, 18.2% vs. 19.3% and 12.5% vs. 8.9%, *p* = 0.538), LPFS (2-year and 5-year, 20.6% vs. 19.8% and 14.2% vs. 10.3%, *p* = 0.647), and DPFS (2-year and 5-year, 28.2% vs. 27.0% and 13.3% vs. 12.3%, *p* = 0.545). Tumor progression was documented in 33/76 (43.4%) patients with cisplatin/vinorelbine and in 49/125 (39.2%) patients with low-dose cisplatin. Locoregional recurrences occurred in 26/76 (34.2%) patients with cisplatin/vinorelbine and in 36/125 (28.8%) patients with low-dose cisplatin. Distant recurrences were documented in 23/76 (30.3%) patients with cisplatin/vinorelbine and in 29/125 patients (23.2%) patients with low-dose cisplatin. It is of valid interest, whether there is a difference in survival when addressing the comparison of chemotherapy protocols after stratification for patient age. Thus, we compared outcomes with cisplatin/vinorelbine vs. low-dose cisplatin separately in elderly (≥70 years) and younger (<70 years) patients. There were no differences in survival (OS, PFS, LPFS, DPFS) in both patient groups (Supplementary Table S6).

## 4. Discussion

Elderly (here defined as ≥70 years) represent a high proportion of lung cancer patients in Europe. In a large patterns of care analysis with >4500 patients, Sant et al. reported that 46.9% were elderly [4]. At the same time, higher age is associated with relevant physiologic changes in the thoracic organs (e.g., reduced chest wall compliance) and further organ systems (e.g., kidney function) [3,5,6,7,8]. It has to be noted that patients with higher ages are less represented in clinical trials [9]. Previous authors highlighted that, especially with focus on development in multimodal treatment of NSCLC, specific strategies are required for elderly patients [12]. Low-dose cisplatin was discussed as an alternative in elderly or patients with relevant comorbidities [18]. Here, we compared clinical characteristics, treatment characteristics, toxicities, and outcomes in younger vs. elderly (≥70 years) patients. Furthermore, we studied prognostic factors in elderly patients. Additionally, we compared clinical outcomes with cisplatin/vinorelbine vs. low-dose cisplatin in definitive RCT, independently of age.

In the presented study, the proportion of males was higher in the elderly group when compared to the younger group (77.8% vs. 67.0%). Comparably, in >3500 patients with concurrent RCT for NSCLC, Stinchcombe et al. found a higher proportion of males in elderly (elderly, 70% vs. younger patients, 62%) [28]. The steepening of the ‘wealth-health gradient‘ could serve as an explanation for these imbalances [29]. Here, physiologically older and more morbid patients are predominantly males [29]. Additionally, in the presented study, elderly patients had a lower Karnofsky index and higher Charlson comorbidity index than younger patients. These aspects were previously reported, e.g., by Zaborowska-Szmit et al. in a study on RCT for NSCLC with the cut-off >65 years [30]. In clinical routine, the Karnofsky index and comorbidities are used for multimodal treatment decisions [31]. Venuta et al. emphasized that the Karnofsky and ECOG scales are of utmost importance, e.g., when assessing patients before surgery [3]. At the same time, the reported data on these scales might serve as an important basis for further development of geriatric assessment tools [32,33].

In the presented study, elderly patients were more often diagnosed in stages IIA-IIIA (vs. IIIB-IIIC) than younger patients. Stinchcombe et al. found comparable differences in stage (stage IIIA vs. IIIB in patients with RCT) [28]. These aspects could possibly be explained by treatment selection. Wang et al. studied >20,000 patients with NSCLC [34]. Patients with local disease underwent surgery less frequently in advanced ages (75–84 years, 50%) than in less advanced ages (65–74 years, 57%) [34]. Concludingly, our study’s differences might reflect treatment patterns at the lung cancer center. Younger age groups in less advanced stages might have been selected for surgery more frequently. Consequently, radiotherapy was possibly not indicated in a certain proportion. Thus, here, less advanced stages are more represented in elderly patients.

Next, we found that bimodality and trimodality treatment (vs. RT only) were less frequently performed in elderly than in younger patients. These findings are supported by a multicenter analysis on routine clinical practice in elderly patients with NSCLC in stages IIIA/IIIB [35]. Cacicedo et al. reported more conservative treatments (e.g., RT alone vs. surgery) in elderly patients [35]. Driessen et al. found that comorbidities and performance status were among the most important reasons for omission of multimodal treatment (here, RCT) [36]. They discussed that elderly patients often undergo less aggressive treatment due to concerns about toxicity risks [36]. In line with these findings, in our cohort, the median Karnofsky index was significantly lower in elderly patients who underwent bimodality and trimodality treatment (vs. RT only) less frequently.

In the presented study, elderly patients experienced less dermatitis and dysphagia (≥grade 1) than younger patients. In this context, it has to be considered that elderly patients were less likely to receive bi- or trimodality therapy vs. RT only. At the same time, RCT is associated with higher rates of esophagitis/dysphagia than RT only [13]. Elderly patients less frequently received cisplatin/vinorelbine when compared to low-dose cisplatin, where previous studies (and, as discussed below, our results) found evidence that low-dose cisplatin is less toxic [18,37]. Additionally, Weiling et al. found evidence for an underestimation of resilience in older patients (here, in a quality of life analysis on different tumor entities) [38]. These aspects could explain the differences in toxicity rates. In the current study, anemia and thrombocytopenia of ≥grade 1 occurred more frequently in elderly patients. The reasons could be physiological changes associated with comorbidities in elderly patients [39]. Aging is generally associated with an increased incidence of anemia [39]. Additionally, anemia has been reported to be associated with chronic obstructive pulmonary disease, which is linked with lung cancer [40,41]. However, in total there were no significant differences between elderly and younger patients in ≥grade 2 or ≥grade 3 toxicities. Thus, treatment with involvement of several modalities seems feasible and without excessive toxicities. This is in line with findings by Dawe et al. [42]. Consequently, older patients should be carefully evaluated and not generally be excluded from multimodal treatment due to concerns about toxicities [12,42].

Knowledge about prognostic factors is relevant for personalized treatment in elderly NSCLC patients, as pointed out by Blanco et al. [43]. In the presented study, females had better outcomes. These findings are in line with the study by Owonikoko et al. on >300,000 elderly patients with lung cancer [44]. May et al. recently addressed these gender-specific differences in a review article [45]. Furthermore, we found better outcomes in patients with higher Karnofsky index (≥80). This emphasizes, beyond age alone, the utmost importance of the index for treatment selection [3,31]. Moreover, multimodal treatment (here, bi- and trimodality vs. RT only) was associated with better outcomes. This parameter remained statistically significant in multivariable survival analysis, along with gender, Karnofsky index, and T stage. Similarly, in elderly patients with NSCLC, Miller et al. found better survival with RCT vs. RT alone [46]. Cacicedo et al. reported that elderly patients with stage IIIA/IIIB treated without surgery (i.e., with concurrent RCT, sequential RT and chemotherapy, or RT only) experience worse outcomes [35]. Taken together, there is evidence for feasibility and good outcomes of multimodal treatment strategies in elderly patients with NSCLC who are deemed fit enough [35,46]. As a strength of our study, patients were treated in the context of the lung cancer center (since 2014, certified by the German Cancer Society). Recent studies highlighted the prognostic value of treatment within this highly standardized setting with involvement of multiple modalities [47]. Walter et al. identified patients with highly individualized tumor board recommendations (i.e., not in accordance with current guidelines) [48]. They emphasized that the need for further studies is on fragile patients (here, advanced age and many comorbidities) [48]. The current study’s results, especially the favorable outcomes with bi- or trimodality treatment in elderly, can be interpreted as an indicator for successful multidisciplinary management in the context of the cancer center.

As part of personalized treatment for elderly or fragile patients in the complete study cohort, low-dose cisplatin was used in 43% of the patients with age ≥70 years, and in 38.3% of the patients with age <70 years. Patients with low-dose cisplatin, compared to patients with cisplatin/vinorelbine, presented with higher age, more comorbidities, and lower N stages. Higher age and more comorbidities reflect clinical routine with the use of low-dose cisplatin especially in elderly or frail patients, as previously proposed [18]. The lower N stages in low-dose cisplatin patients with higher age and more comorbidities might be explained by this study’s design from a radiation oncology point of view. In the context of multidisciplinary management at the cancer center, younger and fitter patients in these N stages might have been more frequently managed by surgery and, thus, radiotherapy might have not been performed. As mentioned above, these findings are supported by the study of Wang et al. that demonstrated the use of surgery less frequently in older patients [34].

We found a remarkable reduction in toxicities with low-dose cisplatin vs. cisplatin/vinorelbine. These findings included dysphagia (≥grades 2 and 3), nausea (≥grades 1 and 2), and leukopenia (≥grades 1, 2, and 3). Furthermore, chemotherapy was completed more frequently in patients with low-dose cisplatin (78.4%) vs. cisplatin/vinorelbine (64.5%). These findings are in line with previous studies, describing a reduced risk of nausea and vomiting and hematologic toxicities with the use of low-dose cisplatin [18,37]. Koning et al. reported higher rates of nausea and vomiting with high-dose vs. low-dose cisplatin in a systematic review [18]. Zazuli et al. found less ≥grade 2 myelotoxicities with daily low-dose cisplatin [37]. Survival outcomes were similar with low-dose cisplatin vs. cisplatin/vinorelbine in the presented study. Koning et al. summarized 13 studies on concurrent RCT (among these, 6 with daily low-dose chemotherapy) and reported 2-year OS between 13% and 38.5% [18]. We found a 2-year OS of 37.9% (cisplatin/vinorelbine) and 33.9% (low-dose cisplatin). Thus, our reported survival rates could be considered in the upper range of these rates. In summary, we found evidence supporting the existing body of literature for low-dose cisplatin as a well-tolerated and acceptably effective alternative option in definitive RCT for elderly or frail patients with NSCLC [18].

Finally, it should be mentioned that the retrospective single-center analysis presented in this study reflects a real-life setting with a representative patient cohort of a large certified lung cancer center. This study focusses on every-day challenges of physicians and health care professionals to provide optimal cancer care, even in elderly and comorbid NSCLC patients. Further prospective (ideally, randomized) clinical trials are needed to define optimal treatment options.

## 5. Conclusions

Identification of the optimal treatment strategy is challenging in elderly (≥70 years) patients with localized NSCLC [12]. Concurrent chemotherapy with low-dose cisplatin represents an option in elderly or frail patients [18]. Here, we present a retrospective single-center study on outcomes (1) in elderly (*n* = 158) vs. younger patients (*n* = 188) and (2), independently of age, in definitive RCT, with low-dose cisplatin (*n* = 125) vs. cisplatin/vinorelbine (*n* = 76). Elderly patients included more males, had a lower Karnofsky index, more comorbidities, and lower prognostic stages. Low-dose cisplatin patients vs. cisplatin/vinorelbine patients had higher age, more comorbidities, and lower prognostic stages. We observed reduced dermatitis and dysphagia and increased anemia and thrombocytopenia in elderly vs. younger patients. No differences in ≥grade 3 toxicities were found. Low-dose cisplatin (compared to cisplatin/vinorelbine) resulted in less toxicities (dysphagia, nausea, and leukopenia) and higher rates of chemotherapy completion. Survival outcomes were lower in elderly vs. younger patients and comparable between low-dose cisplatin and cisplatin/vinorelbine. In elderly patients, gender, Karnofsky index, prognostic stage, and multimodal treatment (including additional surgery/systemic therapy vs. radiotherapy only) were identified as prognostic factors. In conclusion, we found evidence for an acceptable toxicity profile and the need for improvement of outcomes in elderly patients with localized NSCLC. Multimodal strategies (including additional surgery/systemic treatment) showed favorable outcomes and should be taken into consideration in elderly who are deemed fit enough. Low-dose cisplatin should be discussed on an individual basis due to favorable toxicity profile and outcomes.

## Figures and Tables

**Table 1:** Comparison of clinical and treatment characteristics between elderly and younger patients. ^1^ The other histologic findings were as follows: not otherwise specified, *n* = 10, and neuroendocrine tumors, *n* = 3. ^2^ One patient presented with recurrent NSCLC in stage rcT0 rcN3. ^3^ Trimodality therapy: surgery followed by radiochemotherapy, *n* = 7 patients; surgery, followed by chemotherapy and subsequent radiotherapy, *n* = 20 patients; radiochemotherapy, followed by surgery, *n* = 2 patients. Bimodality therapy: radiochemotherapy, *n* = 228 patients; chemotherapy, followed by radiotherapy, *n* = 14 patients; surgery, followed by radiotherapy, *n* = 12 patients. ^4^ *n* = 3, cisplatin/etoposide, *n* = 1, cisplatin/pemetrexed, *n* = 1, carboplatin/paclitaxel, *n* = 1, etoposide/vinorelbine. ^5^ Kruskal–Wallis test. ^6^ Pearson’s chi-square test.

Parameter	≥70 Years, *n* = 158	<70 Years, *n* = 188	*p*-Value
Age (years, median (min–max))	75.8 (70.1–89.2)	61.2 (32.5–69.9)	<0.01 ^5^
Gender			0.02 ^6^
Male (number (%))	123 (77.8)	126 (67.0)	
Female (number (%))	35 (22.2)	62 (33.0)	
Follow-up (months, median, min–max)	11.4 (1.4–153.8)	14.2 (1.2–149.7)	0.04 ^5^
Karnofsky index (median, min–max)	80 (50–90)	90 (20–90)	0.03 ^5^
Charlson comorbidity index (median, min–max)	7 (4–14)	5 (2–12)	<0.01 ^5^
Histology, numbers (%)			0.07 ^5^
Squamous cell carcinoma	100 (63.3)	102 (54.2)	
Adenocarcinoma	54 (34.2)	77 (41.0)	
Other histology ^1^	4 (2.5)	9 (4.8)	
T stage 0–2 ^2^, numbers (%)	52 (32.9)	60 (31.9)	0.84 ^6^
T stage 3–4, numbers (%)	106 (67.1)	128 (68.1)	
N stage 0–1, numbers (%)	48 (30.4)	42 (22.3)	0.08 ^6^
N stage 2–3, numbers (%)	110 (69.6)	146 (77.7)	
UICC stage IIA-IIIA, numbers (%)	89 (56.3)	70 (27.2)	<0.01 ^6^
UICC stage IIIB-IIIC, numbers (%)	69 (43.7)	118 (62.8)	
Treatment concept, numbers (%) ^3^			<0.01 ^5^
Trimodality therapy	10 (6.3)	19 (10.1)	
Bimodality therapy	100 (63.3)	154 (81.9)	
RT only	48 (30.4)	15 (8.0)	
Radiotherapy, applied dose [Gy] (median, min–max)	60 (8–70)	60 (2–70)	0.57 ^5^
Completion of radiotherapy as planned, numbers (%)	129 (81.6)	159 (84.6)	0.47 ^6^
Definitive radiotherapy or radiochemotherapy	144 (91.1)	161 (85.6)	0.11 ^6^
Radiotherapy only	48 (33.3)	15 (9.3)	<0.01 ^6^
Radiochemotherapy	96 (66.7)	146 (90.7)	
Sequential chemotherapy	7 (7.3)	7 (4.8)	0.42 ^6^
Concurrent chemotherapy	89 (92.7)	139 (95.2)	
Cisplatin/vinorelbine	20 (22.5)	62 (44.6)	0.03 ^5^
Low-dose cisplatin	68 (76.4)	72 (51.8)	
Other ^4^	1 (1.1)	5 (3.6)	

**Table 2:** Comparison of clinical and treatment characteristics with cisplatin/vinorelbine vs. low-dose cisplatin in definitive radiochemotherapy (independently of age). ^1^ One patient presented with recurrent NSCLC in stage rcT0 rcN3. ^2^ Kruskal–Wallis test. ^3^ Pearson’s chi-square test.

Parameter	Low-Dose Cisplatin, *n* = 125	Cisplatin/Vinorelbine, *n* = 76	*p*-Value
Age (years, median (min–max))	68.5 (32.5–85.0)	62.6 (39.2–77.8)	<0.01 ^2^
Gender			
Male (number (%))	95 (76.0)	55 (72.4)	0.57 ^3^
Female (number (%))	30 (24.0)	21 (27.6)	
Follow-up (months, median (min–max))	12.3 (1.5–149.7)	9.4 (1.2–60.4)	0.28 ^2^
Karnofsky index (median, min–max)	90 (50–90)	90 (20–90)	0.17 ^2^
Charlson comorbidity index (median, min-max)	6 (2–13)	5 (2–9)	<0.01 ^2^
T stage 0–2 ^1^, numbers (%)	35 (28.0)	25 (32.9)	0.46 ^3^
T stage 3–4, numbers (%)	90 (72.0)	51 (67.1)	
N stage 0–1, numbers (%)	33 (26.4)	7 (9.2)	<0.01 ^3^
N stage 2–3, numbers (%)	92 (73.6)	69 (90.8)	
UICC stage IIB-IIIA, numbers (%)	51 (40.8)	23 (30.2)	0.13 ^3^
UICC stage IIIB-IIIC, numbers (%)	74 (59.2)	53 (69.8)	
Radiotherapy, applied dose [Gy] (median, min-max)	60 (4–66.6)	65 (10–66)	0.37 ^2^
Completion of radiotherapy as planned, numbers (%)	111 (88.8)	60 (78.9)	0.06 ^3^
Completion of concomitant chemotherapy as planned, numbers (%)	98 (78.4)	49 (64.5)	0.03 ^3^

**Table 3:** Comparison of toxicities between elderly and younger patients. Here, Pearson’s chi-square test was used.

Parameter	Elderly Patients (≥70 Years, *n* = 158)	Younger Patients (<70 Years, *n* = 188)	*p*-Value
Dermatitis, ≥grade 1	46 (29.1)	88 (46.8)	<0.01
Dermatitis, ≥grade 2	2 (1.3)	1 (0.5)	0.46
Dysphagia, ≥grade 1	90 (57.0)	130 (69.1)	<0.02
Dysphagia, ≥grade 2	29 (18.4)	48 (25.5)	0.11
Dysphagia, ≥grade 3	8 (5.1)	17 (9.0)	0.15
Nausea, ≥grade 1	45 (28.5)	65 (34.6)	0.23
Nausea, ≥grade 2	12 (7.6)	18 (9.6)	0.51
Pneumonitis, ≥grade 1	43 (27.2)	48 (25.5)	0.72
Pneumonitis, ≥grade 2	19 (12.0)	16 (8.5)	0.28
Pneumonitis, ≥grade 3	4 (2.5)	3 (1.6)	0.54
Lung infection, ≥grade 2	19 (12.0)	29 (15.4)	0.36
Dyspnea, ≥grade 1	108 (68.4)	126 (67.0)	0.79
Dyspnea, ≥grade 2	56 (35.5)	59 (31.4)	0.42
Dyspnea, ≥grade 3	30 (19.0)	24 (12.8)	0.11
Myocardial infarction, ≥grade 2	4 (2.5)	2 (1.1)	0.30
Anemia, ≥grade 1	147 (93.0)	158 (84.0)	0.01
Anemia, ≥grade 2	52 (32.9)	51 (27.1)	0.24
Anemia, ≥grade 3	12 (7.6)	13 (6.9)	0.81
Leukopenia, ≥grade 1	86 (54.4)	107 (56.9)	0.64
Leukopenia, ≥grade 2	54 (34.2)	69 (36.7)	0.63
Leukopenia, ≥grade 3	31 (19.6)	39 (20.7)	0.80
Thrombocytopenia, ≥grade 1	80 (50.6)	73 (38.8)	0.03
Thrombocytopenia, ≥grade 2	18 (11.4)	15 (8.0)	0.28
Thrombocytopenia, ≥grade 3	7 (4.4)	7 (3.7)	0.74

**Table 4:** Comparison of toxicities with cisplatin/vinorelbine vs. low-dose cisplatin in definitive radiochemotherapy (independently of age). Here, Pearson’s chi-square test was used.

Parameter	Low-Dose Cisplatin, *n* = 125	Cisplatin/Vinorelbine, *n* = 76	*p*-Value
Dermatitis, ≥grade 1	51 (40.8)	31 (40.8)	1.00
Dermatitis, ≥grade 2	1 (0.8)	0 (0.0)	0.43
Dysphagia, ≥grade 1	78 (62.4)	51 (67.1)	0.50
Dysphagia, ≥grade 2	24 (19.2)	24 (31.6)	<0.05
Dysphagia, ≥grade 3	6 (4.8)	11 (14.4)	0.02
Nausea, ≥grade 1	33 (26.4)	32 (42.1)	0.02
Nausea, ≥grade 2	5 (4.0)	12 (15.8)	<0.01
Pneumonitis, ≥grade 1	33 (26.4)	15 (19.7)	0.28
Pneumonitis, ≥grade 2	13 (10.4)	4 (5.3)	0.20
Pneumonitis, ≥grade 3	2 (1.6)	0 (0.0)	0.27
Lung infection, ≥grade 2	21 (16.8)	11 (14.5)	0.66
Dyspnea, ≥grade 1	79 (63.2)	48 (63.2)	1.00
Dyspnea, ≥grade 2	41 (32.8)	22 (29.0)	0.57
Dyspnea, ≥grade 3	20 (16.0)	7 (9.2)	0.17
Myocardial infarction, ≥grade 2	2 (1.6)	0 (0.0)	0.27
Anemia, ≥grade 1	108 (86.4)	67 (88.2)	0.72
Anemia, ≥grade 2	34 (27.2)	22 (28.9)	0.79
Anemia, ≥grade 3	7 (5.6)	7 (9.2)	0.33
Leukopenia, ≥grade 1	78 (62.4)	58 (76.3)	0.04
Leukopenia, ≥grade 2	45 (36.0)	47 (61.8)	<0.01
Leukopenia, ≥grade 3	18 (14.4)	31 (40.8)	<0.01
Thrombocytopenia, ≥grade 1	69 (55.2)	34 (44.7)	0.15
Thrombocytopenia, ≥grade 2	11 (8.8)	9 (11.8)	0.52
Thrombocytopenia, ≥grade 3	2 (1.6)	4 (5.3)	0.14

**Table 5:** Prognostic factors in elderly patients (≥70 years), multivariable Cox regression analysis. OS—overall survival. PFS—progression-free survival. LPFS—locoregional progression-free survival. DPFS—distant progression-free survival. HR—hazard ratio. CI—confidence interval. RT—radiotherapy.

Parameter (Numbers of Patients)	OS		PFS		LPFS		DPFS	
	HR (95% CI)	*p*-Value	HR (95% CI)	*p*-Value	HR (95% CI)	*p*-Value	HR (95% CI)	*p*-Value
Gender (female, 35; male, 123)	0.60 (0.37–0.97)	0.04	0.61 (0.39–0.95)	0.03	0.60 (0.39–0.94)	0.03	0.6 (0.38–0.96)	0.03
T stage (T0–2, 52; T3–4, 106)	1.53 (1.03–2.27)	0.04	1.47 (1.01–2.13)	0.04	1.41 (0.97–2.06)	0.07	1.47 (1.00–2.15)	<0.05
Histology (adenocarcinoma, 54; other histology, 104)	0.90 (0.60–1.37)	0.64	0.87 (0.58–1.29)	0.48	0.82 (0.55–1.22)	0.33	0.92 (0.61–1.38)	0.68
Treatment concept, RT only (48) vs. bi- and trimodality therapy (110)	1.66 (1.12–2.46)	0.01	1.69 (1.17–2.44)	<0.01	1.67 (1.15–2.41)	<0.01	1.51 (1.04–2.21)	0.03
Karnofsky index, ≥median (75) vs. <median (83), median = 80	0.56 (0.39–0.81)	<0.01	0.64 (0.45–0.9)	0.01	0.6 (0.42–0.85)	<0.01	0.59 (0.41–0.84)	<0.01

## Data Availability

The datasets generated during and/or analyzed during the current study are available from the corresponding author on reasonable request.

## Supplementary Materials

The following supporting information can be downloaded at: https://www.mdpi.com/article/10.3390/cancers16020327/s1, Supplementary Table S1. Comparison of clinical and treatment characteristics between patients treated with definitive radiotherapy (RT) only, definitive radiochemotherapy with low-dose cisplatin, and definitive radiochemotherapy with cisplatin/vinorelbine. The statistical comparisons were performed with chi-square test or Kruskal–Wallis test. Supplementary Table S2. Comparison of toxicities between patients who received definitive radiotherapy (RT) only, definitive radiochemotherapy with low-dose cisplatin, and definitive radiochemotherapy with cisplatin/vinorelbine. Here, Pearson’s chi-square test was used. Supplementary Table S3. Prognostic factors in all eligible patients (total, *n* = 346, patients ≥ 70 years, *n* = 158, and patients < 70 years, *n* = 188). Univariable Cox regression analysis. OS—overall survival. PFS—progression-free survival. LPFS—locoregional progression-free survival. DPFS—distant progression-free survival. HR—hazard ratio. CI—confidence interval. RT—radiotherapy. Supplementary Table S4. Prognostic factors in all eligible patients (total, *n* = 346, patients ≥ 70 years, *n* = 158, and patients < 70 years, *n* = 188). Multivariable Cox regression analysis. OS—overall survival. PFS—progression-free survival. LPFS—locoregional progression-free survival. DPFS—distant progression-free survival. HR—hazard ratio. CI—confidence interval. RT—radiotherapy. Supplementary Table S5. Prognostic factors in elderly patients (≥70 years, *n* = 158). Univariable Cox regression analysis. OS—overall survival. PFS—progression-free survival. LPFS—locoregional progression-free survival. DPFS—distant progression-free survival. HR—hazard ratio. CI—confidence interval. RT—radiotherapy. Supplementary Table S6. Survival (univariable Cox regression analysis) in patients who received definitive radiochemotherapy with cisplatin/vinorelbine vs. low-dose cisplatin (stratified by age). OS—overall survival. PFS—progression-free survival. LPFS—locoregional progression-free survival. DPFS—distant progression-free survival. HR—hazard ratio. CI—confidence interval. RT—radiotherapy.
