# Supplementary material for: Outcomes of Multimodal Treatment in Elderly Patients with Localized Non-Small Lung Cancer from a Radiation Oncology Point of View: Special Focus on Low-Dose Cisplatin
Source: Cancers (Basel). 2024 Jan 11;16(2):327. doi: 10.3390/cancers16020327 (PMC10814481; doi:10.3390/cancers16020327)
Supplement: Supplementary file 1 [file cancers-16-00327-s001.zip › Supplementary Table S5.pdf]

Table S5. Prognostic factors in elderly patients (≥70 years, n = 158). Univariable Cox regression analysis. OS—overall survival. PFS—progression-free survival. LPFS—locoregional progression-free survival. DPFS—distant progression-free survival. HR—hazard ratio. CI—confidence interval. RT—radiotherapy.

Parameter (numbers of patients)	OS		PFS		LPFS		DPFS	
	HR (95% CI)	p-value	HR (95% CI)	p-value	HR (95% CI)	p-value	HR (95% CI)	p-value
**Gender (female, 35; male, 123)**	0.53 (0.33 - 0.85)	<0.01	0.54 (0.35 - 0.84)	<0.01	0.55 (0.35 - 0.85)	<0.01	0.54 (0.35 - 0.86)	<0.01
**T stage (T0-2, 52; T3-4, 106)**	1.51 (1.02 - 2.23)	0.04	1.52 (1.05 - 2.19)	0.03	1.47 (1.02 - 2.13)	0.04	1.47 (1.01 - 2.14)	<0.05
**N stage (N0-1, 48; N2-3, 110)**	0.92 (0.63 - 1.36)	0.68	0.97 (0.68 - 1.4)	0.88	0.99 (0.69 - 1.44)	0.97	0.94 (0.65 - 1.37)	0.76
**Histology (adenocarcinoma, 54; other histology, 104)**	0.67 (0.45 - 0.99)	0.04	0.64 (0.44 - 0.93)	0.02	0.62 (0.43 - 0.9)	0.01	0.68 (0.47 - 1)	<0.05
**Treatment concept**								
**RT only (48) vs. bimodality therapy (100)**	1.55 (1.05 - 2.28)	0.03	1.53 (1.07 - 2.21)	0.02	1.53 (1.06 - 2.21)	0.02	1.41 (0.97 - 2.05)	0.07
**RT only (48) vs. trimodality therapy (10)**	7.8 (2.33 - 26.11)	<0.01	7.57 (2.63 - 21.77)	<0.01	7.46 (2.59 - 21.49)	<0.01	5.98 (2.07 - 17.26)	<0.01
**RT only (48) vs. bi- and trimodality therapy (110)**	1.78 (1.21 - 2.61)	<0.01	1.78 (1.24 - 2.55)	<0.01	1.77 (1.23 - 2.55)	<0.01	1.62 (1.12 - 2.35)	0.01
**Bimodality therapy (100) vs. trimodality therapy (10)**	3.86 (1.40 – 10.64)	<0.01	3.23 (1.39 – 7.51)	<0.01	3.21 (1.38 – 7.46)	<0.01	3.56 (1.42 – 8.90)	<0.01
**Karnofsky index, ≥median (75) vs. <median (83)**	0.57 (0.4 - 0.83)	<0.01	0.63 (0.45 - 0.89)	<0.01	0.6 (0.43 - 0.85)	<0.01	0.6 (0.42 - 0.86)	<0.01
**Radiotherapy, applied dose, >60 Gy (67) vs. ≤ 60 Gy (91)**	0.77 (0.53 – 1.12)	0.18	0.76 (0.54 - 1.08)	0.13	0.81 (0.57 – 1.15)	0.24	0.72 (0.51 – 1.03)	0.07
