# Supplementary material for: Outcomes of Multimodal Treatment in Elderly Patients with Localized Non-Small Lung Cancer from a Radiation Oncology Point of View: Special Focus on Low-Dose Cisplatin
Source: Cancers (Basel). 2024 Jan 11;16(2):327. doi: 10.3390/cancers16020327 (PMC10814481; doi:10.3390/cancers16020327)
Supplement: Supplementary file 1 [file cancers-16-00327-s001.zip › Supplementary Table S6.pdf]

Table S6. Survival (univariable Cox regression analysis) in patients who received definitive radiochemotherapy with cisplatin/vinorelbine vs. low-dose cisplatin (stratified by age). OS—overall survival. PFS—progression-free survival. LPFS—locoregional progression-free survival. DPFS—distant progression-free survival. HR—hazard ratio. CI—confidence interval. RT—radiotherapy.

Parameter (numbers of patients)	OS		PFS		LPFS		DPFS	
	HR (95% CI)	p-value	HR (95% CI)	p-value	HR (95% CI)	p-value	HR (95% CI)	p-value
**Cisplatin/vinorelbine vs. low-dose cisplatin, only patients <70 years (125, cisplatin/vinorelbine, 58, low-dose cisplatin, 67)**	0.92 (0.58-1.46)	0.73	0.97 (0.66-1.44)	0.89	0.98 (0.65-1.48)	0.93	0.93 (0.61-1.41)	0.72
**Cisplatin/vinorelbine vs. low-dose cisplatin, only patients ≥70 years (76, cisplatin/vinorelbine, 18, low-dose cisplatin, 58)**	0.79 (0.42-1.51)	0.48	0.76 (0.42-1.38)	0.37	0.73 (0.40-1.32)	0.30	0.72 (0.39-1.31)	0.28
