# Supplementary material for: Outcomes of Multimodal Treatment in Elderly Patients with Localized Non-Small Lung Cancer from a Radiation Oncology Point of View: Special Focus on Low-Dose Cisplatin
Source: Cancers (Basel). 2024 Jan 11;16(2):327. doi: 10.3390/cancers16020327 (PMC10814481; doi:10.3390/cancers16020327)
Supplement: Supplementary file 1 [file cancers-16-00327-s001.zip › Supplementary Table S1.pdf]

Table S1. Comparison of clinical and treatment characteristics between patients treated with definitive radiotherapy (RT) only, definitive radiochemotherapy with low-dose cisplatin, and definitive radiochemotherapy with cisplatin/vinorelbine. The statistical comparisons were performed with Chi-square test or Kruskal–Wallis test.

Parameter	RT only, n=58	Low-dose cisplatin, n=125	Cisplatin/ vinorelbine, n=76	Comparison of RT only vs. low-dose cisplatin, p-value	Comparison of RT only vs. cisplatin/ vinorelbine, p-value
Age (years, median (min-max))	77.1 (56.5 – 89.2)	68.5 (32.5 – 85.0)	62.6 (39.2 – 77.8)	<0.01	<0.01
Gender					
Male (number (%))	46 (79.3)	95 (76.0)	55 (72.4)	0.62	0.36
Female (number (%))	12 (20.7)	30 (24.0)	21 (27.6)		
Follow-up (months, median, min-max)	8.7 (1.5 – 58.1)	12.3 (1.5 – 149. 7)	9.4 (1.2 – 60.4)	0.05	0.36
Karnofsky index (median, min-max)	80 (50 – 90)	90 (50 – 90)	90 (20 – 90)	<0.01	<0.01
Charlson Comorbidity Index (median, min-max)	7.5 (3-14)	6 (2-13)	5 (2-9)	<0.01	<0.01
T stage 0-2, numbers (%)	23 (39.7)	35 (28.0)	25 (32.9)	0.11	0.42
T stage 3-4, numbers (%)	35 (60.3)	90 (72.0)	51 (67.1)		
N stage 0-1, numbers (%)	16 (27.6)	33 (26.4)	7 (9.2)	0.87	0.01
N stage 2-3, numbers (%)	42 (72.4)	92 (73.6)	69 (90.8)		
UICC stage IIB-IIIA, numbers (%)	32 (55.2)	51 (40.8)	23 (30.2)	0.07	0.02
UICC stage IIIB-IIIC, numbers (%)	26 (44.8)	74 (59.2)	53 (69.8)		
Radiotherapy, applied dose [Gy] (median, min-max)	60 (2 – 66)	60 (4 – 66.6)	65 (10 – 66)	0.04	0.02
Completion of radiotherapy as planned, numbers (%)	42 (72.4)	111 (88.8)	60 (78.9)	<0.01	0.38
