# Supplementary material for: Outcomes of Multimodal Treatment in Elderly Patients with Localized Non-Small Lung Cancer from a Radiation Oncology Point of View: Special Focus on Low-Dose Cisplatin
Source: Cancers (Basel). 2024 Jan 11;16(2):327. doi: 10.3390/cancers16020327 (PMC10814481; doi:10.3390/cancers16020327)
Supplement: Supplementary file 1 [file cancers-16-00327-s001.zip › Supplementary Table S2.pdf]

Table S2. Comparison of toxicities between patients who received definitive radiotherapy (RT) only, definitive radiochemotherapy with low-dose cisplatin, and definitive radiochemotherapy with cisplatin/vinorelbine. Here, Pearson's Chi-square test was used.

Parameter	RT only, n=58	Low-dose cisplatin, n=125	Cisplatin/ vinorelbine, n=76	Comparison of RT only vs. low-dose cisplatin, p-value	Comparison of RT only vs. cisplatin/ vinorelbine, p-value
Dermatitis, ≥grade 1	16 (27.6)	51 (40.8)	31 (40.8)	0.08	0.11
Dermatitis, ≥grade 2	1 (1.7)	1 (0.8)	0 (0.0)	0.58	0.25
Dysphagia, ≥grade 1	32 (55.2)	78 (62.4)	51 (67.1)	0.35	0.16
Dysphagia, ≥grade 2	9 (15.5)	24 (19.2)	24 (31.6)	0.55	0.03
Dysphagia, ≥grade 3	2 (3.4)	6 (4.8)	11 (14.4)	0.68	0.03
Nausea, ≥grade 1	11 (19.0)	33 (26.4)	32 (42.1)	0.27	<0.01
Nausea, ≥grade 2	5 (8.6)	5 (4.0)	12 (15.8)	0.20	0.22
Pneumonitis, ≥grade 1	16 (27.6)	33 (26.4)	15 (19.7)	0.87	0.29
Pneumonitis, ≥grade 2	9 (15.5)	13 (10.4)	4 (5.3)	0.32	<0.05
Pneumonitis, ≥grade 3	3 (5.2)	2 (1.6)	0 (0.0)	0.17	0.04
Lung infection, ≥grade 2	5 (8.6)	21 (16.8)	11 (14.5)	0.14	0.30
Dyspnea, ≥grade 1	44 (75.9)	79 (63.2)	48 (63.2)	0.09	0.12
Dyspnea, ≥grade 2	26 (44.9)	41 (32.8)	22 (29.0)	0.11	0.06
Dyspnea, ≥grade 3	16 (27.6)	20 (16.0)	7 (9.2)	0.07	<0.01
Myocardial infarction, ≥grade 2	3 (5.2)	2 (1.6)	0 (0.0)	0.17	0.04
Anemia, ≥grade 1	52 (89.7)	108 (86.4)	67 (88.2)	0.54	0.79
Anemia, ≥grade 2	22 (37.9)	34 (27.2)	22 (28.9)	0.14	0.27
Anemia, ≥grade 3	8 (13.8)	7 (5.6)	7 (9.2)	0.06	0.40
Leukopenia, ≥grade 1	13 (22.4)	78 (62.4)	58 (76.3)	<0.01	<0.01
Leukopenia, ≥grade 2	4 (6.9)	45 (36.0)	47 (61.8)	<0.01	<0.01
Leukopenia, ≥grade 3	2 (3.4)	18 (14.4)	31 (40.8)	0.03	<0.01
Thrombocytopenia, ≥grade 1	15 (25.9)	69 (55.2)	34 (44.7)	<0.01	0.02
Thrombocytopenia, ≥grade 2	3 (5.2)	11 (8.8)	9 (11.8)	0.39	0.20
Thrombocytopenia, ≥grade 3	2 (3.4)	2 (1.6)	4 (5.3)	0.43	0.61
