# Supplementary material for: Outcomes of Multimodal Treatment in Elderly Patients with Localized Non-Small Lung Cancer from a Radiation Oncology Point of View: Special Focus on Low-Dose Cisplatin
Source: Cancers (Basel). 2024 Jan 11;16(2):327. doi: 10.3390/cancers16020327 (PMC10814481; doi:10.3390/cancers16020327)
Supplement: Supplementary file 1 [file cancers-16-00327-s001.zip › Supplementary Table S3.pdf]

Table S3. Prognostic factors in all eligible patients (total, n = 346, patients ≥70 years, n = 158, and patients <70 years, n = 188). Univariable Cox regression analysis. OS—overall survival. PFS—progression-free survival. LPFS—locoregional progression-free survival. DPFS—distant progression-free survival. HR—hazard ratio. CI—confidence interval. RT—radiotherapy.

**Parameter (numbers of patients)**	**OS**		**PFS**		**LPFS**		**DPFS**	
	**HR (95% CI)**	**p-value**	**HR (95% CI)**	**p-value**	**HR (95% CI)**	**p-value**	**HR (95% CI)**	**p-value**
**Gender (female, 97; male, 249)**	0.65 (0.48-0.87)	<0.01	0.63 (0.48-0.82)	<0.01	0.60 (0.45-0.79)	<0.01	0.69 (0.52-0.91)	<0.01
**Age (≥70 years, n=158, <70 years, n=188)**	1.44 (1.11-1.85)	<0.01	1.09 (0.86-1.37)	0.49	1.20 (0.95-1.52)	0.13	1.17 (0.92-1.49)	0.2
**Karnofsky Index, median=90, ≥median (187) vs. <median (159)**	0.67 (0.52-0.86)	<0.01	0.74 (0.59-0.93)	0.01	0.72 (0.57-0.92)	<0.01	0.74 (0.58-0.95)	0.02
**Charlson Comorbidity Index, median=6, ≥median (208) vs. <median (138)**	1.45 (1.11-1.90)	<0.01	1.12 (0.88-1.42)	0.36	1.23 (0.96-1.57)	0.10	1.18 (0.92-1.53)	0.19
**T stage (T0-2, 112; T3-4, 234)**	1.43 (1.08-1.89)	0.01	1.38 (1.07-1.77)	0.01	1.33 (1.03-1.72)	0.03	1.36 (1.05-1.77)	0.02
**N stage (N0-1, 90; N2-3, 256)**	1.19 (0.88-1.60)	0.26	1.20 (0.92-1.56)	0.19	1.20 (0.91-1.57)	0.20	1.24 (0.94-1.65)	0.13
**Histology (adenocarcinoma, 131; other histology, 215)**	0.78 (0.59-1.01)	0.06	0.81 (0.64-1.03)	0.09	0.75 (0.59-0.96)	0.02	0.87 (0.67-1.11)	0.25
**Treatment concept**								
**RT only (63) vs. bimodality therapy (254)**	1.48 (1.08-2.04)	0.01	1.25 (0.94-1.67)	0.13	1.34 (1.0-1.80)	0.051	1.23 (0.95-1.73)	0.11
**RT only (63) vs. trimodality therapy (29)**	3.95 (2.15-7.27)	<0.01	4.36 (2.46-7.71)	<0.01	4.51 (2.54-8.02)	<0.01	4.09 (2.26-7.40)	<0.01
**RT only (63) vs. bi- and trimodality therapy (283)**	1.74 (1.27-2.37)	<0.01	1.51 (1.13-2.02)	<0.01	1.63 (1.22-2.18)	<0.01	1.52 (1.13-2.05)	<0.01
**Bimodality therapy (254) vs. trimodality therapy (29)**	0.37 (0.22-0.64)	<0.01	0.32 (0.20-0.52)	<0.01	0.33 (0.20-0.54)	<0.01	0.33 (0.19-0.55)	<0.01
**RT, applied dose, >60 Gy (135) vs. ≤ 60 Gy (211)**	0.82 (0.63-1.07)	0.14	0.84 (0.66-1.06)	0.15	0.88 (0.69-1.12)	0.30	0.80 (0.63-1.03)	0.08
