# Supplementary material for: Outcomes of Multimodal Treatment in Elderly Patients with Localized Non-Small Lung Cancer from a Radiation Oncology Point of View: Special Focus on Low-Dose Cisplatin
Source: Cancers (Basel). 2024 Jan 11;16(2):327. doi: 10.3390/cancers16020327 (PMC10814481; doi:10.3390/cancers16020327)
Supplement: Supplementary file 1 [file cancers-16-00327-s001.zip › Supplementary Table S4.pdf]

Table S4. Prognostic factors in all eligible patients (total, n = 346, patients ≥70 years, n = 158, and patients <70 years, n = 188). Multivariable Cox regression analysis. OS—overall survival. PFS—progression-free survival. LPFS—locoregional progression-free survival. DPFS—distant progression-free survival. HR—hazard ratio. CI—confidence interval. RT—radiotherapy.

Parameter (numbers of patients)	OS		PFS		LPFS		DPFS	
	HR (95% CI)	p-value	HR (95% CI)	p-value	HR (95% CI)	p-value	HR (95% CI)	p-value
Gender (female, 97; male, 249)	0.71 (0.52-0.96)	0.03	0.66 (0.50-0.87)	<0.01	0.64 (0.48-0.84)	<0.01	0.73 (0.55-0.96)	0.03
Age (≥70 years, n=158, <70 years, n=188)	1.35 (1.04-1.76)	0.03	1.05 (0.80-1.39)	0.72	1.12 (0.87-1.42)	0.38	1.10 (0.86-1.42)	0.44
Karnofsky Index, median=90, ≥median (187) vs. <median (159)	0.71 (0.54-0.92)	<0.01	0.78 (0.62-1.00)	0.046	0.77 (0.61-0.99)	0.04	0.78 (0.61-1.00)	0.047
Charlson Comorbidity Index, median=6, ≥median (208) vs. <median (138)	1.08 (0.78-1.51)	0.64	0.96 (0.74-1.23)	0.73	0.97 (0.71-1.31)	0.82	0.98 (0.72-1.32)	0.87
T stage (T0-2, 112; T3-4, 234)	1.44 (1.09-1.91)	0.01	1.35 (1.05-1.74)	0.02	1.29 (1.00-1.67)	0.05	1.35 (1.04-1.76)	0.03
Histology (adenocarcinoma, 131; other histology, 215)	0.92 (0.70-1.21)	0.56	0.95 (0.74-1.22)	0.70	0.86 (0.67-1.12)	0.26	1.01 (0.78-1.31)	0.95
Treatment concept, RT only (63) vs. bi- and trimodality therapy (283)	1.44 (1.03-2.00)	0.03	1.37 (1.02-1.85)	0.04	1.46 (1.08-1.97)	0.01	1.41 (1.04-1.91)	0.03
